# Quality of life of COVID-19 recovered patients in Bangladesh

**DOI:** 10.1371/journal.pone.0257421

**Published:** 2021-10-13

**Authors:** Mohammad Delwer Hossain Hawlader, Md. Utba Rashid, Md. Abdullah Saeed Khan, Tasnim Ara, Mohammad Hayatun Nabi, Miah Md. Akiful Haque, Kazi Farhana Matin, Mohammad Ali Hossain, Mahfil Ara Rahman, Mosharop Hossian, Shuvajit Saha, Ridwana Maher Manna, Md. Yeasin Arafat, Sabrina Yesmin Barsha, Ramisha Maliha, Jeba Zaman Khan, Soumik Kha, S. M. Rezwanul Hasan, Mehedi Hasan, Saleka Raihana Siddiquea, Joyeeta Khan, A. M. Khairul Islam, Rubaya Rashid, Naima Nur, Omar Khalid, Fatiha Bari, Mohammad Lutfor Rahman

**Affiliations:** 1 Department of Public Health, North South University, Dhaka, Bangladesh; 2 Nutrition and Clinical Services Division (NCSD), International Centre for Diarrhoeal Disease Research, Bangladesh (ICDDR, B), Mohakhali, Dhaka, Bangladesh; 3 Pi Research Consultancy Center, Lalbagh, Dhaka, Bangladesh; 4 Institute of Statistical Research and Training (ISRT), University of Dhaka, Dhaka, Bangladesh; 5 Ibn Sina Medical College Hospital, Kallyanpur, Dhaka, Bangladesh; 6 Centre for Injury Prevention & Research Bangladesh, Mohakhali, Dhaka, Bangladesh; 7 Projahnmo Research Foundation, Dhaka, Bangladesh; University of Southern Queensland, AUSTRALIA

## Abstract

Coronavirus Disease-2019 (COVID-19) quickly surged the whole world and affected people’s physical, mental, and social health thereby upsetting their quality of life. Therefore, we aimed to investigate the quality of life (QoL) of COVID-19 positive patients after recovery in Bangladesh. This was a study of adult (aged ≥18 years) COVID-19 individuals from eight divisions of Bangladesh diagnosed and confirmed by Reverse Transcription-Polymerase Chain Reaction (RT-PCR) from June 2020 to November 2020. Given a response rate of 60% in a pilot study, a random list of 6400 COVID-19 patients was generated to recruit approximately 3200 patients from eight divisions of Bangladesh and finally a total of 3244 participants could be recruited for the current study. The validated Bangla version of the World Health Organization Quality of Life Brief (WHOQOL-BREF) questionnaire was used to assess the QoL. Data were analyzed by STATA (Version 16.1) and R (Version 4.0.0). All the procedures were conducted following ethical approval and in accordance with the Declaration of Helsinki. The mean scores of QoL were highest for the physical domain (68.25±14.45) followed by social (65.10±15.78), psychological (63.28±15.48), and environmental domain (62.77±13.07). Psychological and physical domain scores among females were significantly lower than the males (*p<*0.001). The overall quality of life was lower in persons having a chronic disease. Participants over 45 years of age were 52% less likely to enjoy good physical health than the participants aged below 26 years (AOR: 0.48, CI: 0.28–0.82). The quality of life of employed participants was found 1.8 times higher than the unemployed (AOR: 1.80, CI: 1.11–2.91). Those who were admitted to hospitals during infection had a low QoL score in physical, psychological, and socials domains. However, QoL improved in all aspect except the psychological domain for each day passed after the diagnosis. These findings call for a focus on the quality of life of the COVID-19 affected population, with special emphasis given to females, older adults, unemployed, and people with comorbidities.

## Introduction

The Coronavirus Disease-2019 (COVID-19) pandemic wrecked the world with over 100 million confirmed cases and more than 2 million deaths till vaccination got started [1]. It appeared as a severe infectious disease and impacted all ages and sexes, especially older adults with comorbidities [2, 3]. Numerous symptoms and complications are associated with it, which may include but are not limited to sepsis, multi-organ failure, and eventually death [4–7]. Apart from physical health it has also affected mental health causing significant anxiety and depression among people [8] and have affected day-to-day lives, jobs, and relationships. Hence, COVID-19 has remarkably affected people’s quality of life irrespective of them being infected or not [9, 10].

The World Health Organization (WHO) defined quality of life as an individual’s perception of their position in life in the context of the culture and value systems in which they live, and concerning their goals, expectations, standards, and concerns [11]. COVID-19 has been found to cause significant physical and psychological impairment [12] leading to decreased health-related quality of life (HRQoL) [9]. Studies showed that all domains quality of life of people were affected during the lockdown period [13]. However, most of these studies were conducted to assess the pandemic’s effect among general people. There is a glaring dearth of literature where the quality of life (QoL) is explored among the patients recovered from COVID-19, especially in Bangladesh. Therefore, we aimed to conduct a QoL assessment of COVID-19 patients after recovery in eight divisions of Bangladesh.

## Methods and materials

### Study design and participants

This was a nationwide study of COVID-19 patients who were diagnosed and confirmed by Reverse Transcription-Polymerase Chain Reaction (RT-PCR) from June 2020 to November 2020, subsequently recovered either clinically or by a negative RT-PCR. Clinical recovery was defined as passing 14 days after initial positive test for asymptomatic patients, complete absence of fever, cough, shortness of breath for at least 3 days for mild to moderate pneumonia patients, and discharge with advice from the hospital for severe or critical patients. Persons who were currently being treated for COVID-19, children (<18 years), pregnant women and critically ill were excluded from the study. The list of available COVID-19 positive patients was obtained, after formal written approval, from Civil Surgeon’s (CS) offices of the relevant districts of all eight divisions of Bangladesh. According to the Institute of Epidemiology and Disease Control and Research (IEDCR), a total of 302907 patients had been tested positive until 4November 2020 in Bangladesh. Among them 167499 in Dhaka division (level two administrative zone of Bangladesh), 48785 in Chattogram, 21126 in Rajshahi, 12947 in Rangpur, 22990 in Khulna, 6784 in Mymensingh, 13758 in Sylhet, and 9018 in Barisal divisions [14]. A research team consisting of 27 members prepared a structured questionnaire. We conducted a pilot survey on 100 post COVID-19 individuals selected randomly from the sampling frame to test the questionnaire. We found that the response rate was around 60% (due to call drop, call waiting, inactive number, network problem, and refusal to give an interview). From the opinion of statistical experts, we intended to interview at least 200 post COVID-19 patients from each of the urban and rural stratums (16 stratums for 8 divisions) and thus intended sample size appeared to be a total of 3200 patients which was roughly 1% of the total population of COVID-19 patients for 8 divisions. However, as there was no prior information about residence status, whether the patients were from urban or rural, of the COVID-19 patients, simply we targeted to collect information on 400 patients from each division. As the pilot survey, conducted with a view to testing the questionnaire, showed that there around 60% non-response, we increased our target sample to 6400 to reach our goal of 3200 patients. A list of randomly selected COVID-19 positive patients (where randomization was accomplished through statistical programming software R version 4.0.0) from each of the divisions was supplied to the data collection team. The data collection team could approach 4584 patients because 1332 individuals declined to participate, 466 individuals did not receive the calls, and 18 patients were dead at the time of interview, hence, were excluded ultimately. Thus, the data collection team successfully completed the interview of 3244 COVID-19 recovered patients. The survey was conducted from 16 November 2020 to 17 January 2021 including the pilot testing.

### Study instrument

All the COVID-19 recovered patients in the sample underwent interviews by the pre-formed and pre-tested questionnaire consisting of socio-demographic information, personal history, symptoms and comorbidity profile, and QoL assessment. The QoL part was adapted from the 26-item World Health Organization (WHO) endorsed quality of life questionnaire (brief version), known as WHOQOL-BREF [11]. The validated Bangla version [15] of WHOQOL-BREF was used with permission from original authors.

#### Sociodemographic profile

The sociodemographic section of the questionnaire obtained information regarding patients address, age, sex, residence, religion, highest level of education, occupation, marital status, monthly income, and if the participant is healthcare worker or not.

#### Personal history, comorbidity and symptom profile

The second section comprised of questions related to patient’s admission history in hospital due to COVID-19, smoking history, comorbidity profile including hypertension, diabetes, heart disease, asthma/COPD, chronic kidney disease (CKD) and cancer, and a list of symptoms that might occur or persists after COVID-19.

#### WHOQOL-BREF

We used WHOQOL-BREF to measure the QoL of COVID-19 positive patients, which is a validated short version of the WHOQOL-100 quality of life assessment tool [16]. The later instrument was developed by the WHOQOL Group with fifteen international field centers, simultaneously, in an attempt to develop a QoL assessment that would be applicable cross-culturally. All items in the WHOQOL-BREF are rated on a 5-point scale and encompasses four domains of QoL, namely, the physical, psychological, social relationships, and environment domains. The WHOQOL-BREF is the most widely used generic QoL assessment tool across the globe. This moderately sized (25 items) instrument covers the whole range of QoL deficits, has a simple response format, but allows fine-grained discrimination of QoL across individuals. Considering the points to be noted during a cross-sectional assessment of QoL [17], we found that WHOQOL-BREF best fits our purpose. Hence, the instrument was adapted for our study.

### Study procedure

As our study participants were from the whole of the country, we assigned our interviewers based on their locality to avoid the language barrier. We conducted the interview over the phone considering the current pandemic situation. Before interview we explained the respondents that there is no right or wrong answers. Misunderstood items were simply repeated, and respondents were encouraged to interpret the questions in their own way. The scoring of the WHOQOL-BREF part of the questionnaire was done in accordance with the manual [11]. The WHOQOL-BREF showed a good internal consistency among our respondents (Cronbach’s alpha coefficient = 0.89).

### Statistical analysis

We applied descriptive and inferential methods to determine the impact of COVID-19 on QoL as a whole as well as in four domains, namely physical, psychological, social, and environmental. Normality assumption was checked and then, analysis of variance (ANOVA) models were employed to compare continuous variables for different groups. Categorical variables were described by frequencies (percent), and chi-square tests were used to identify associations between groups. Independent sample t-test was used for continuous variables when comparing means of two groups. All tests were two-tailed and p-values less than or equal to 0.05 were considered statistically significant. The internal consistency of QoL scores, measured by the WHOQOL-BREF instrument, was checked by Cronbach’s alpha coefficient. QoL scores were transformed into binary scores by considering a score greater or equal to 50 as 1 (“good”), otherwise as 0 (“poor”) to implement a binary logistic regression whereby identifying influencing factors for quality of life. A receiver operating characteristic (ROC) curve was used to illustrate the diagnostic ability of the binary classifier system with its varying discrimination threshold. We used Statistical software STATA (Version 16.1) and R (Version 4.0.0) for statistical analysis.

### Ethical consideration

Ethical approval for this study was obtained from the ethical review committee (ERC)/institutional review board (IRB), North South University (2020/OR-NSU/IRB-No.0801). All the procedures were conducted following the ethical standards laid down in the 1964 Declaration of Helsinki and its later amendments or comparable ethical standards wherever applicable. During the telephonic interview, verbal informed consent was obtained from all the participants before inclusion. After receiving the call participants were informed about the purpose of the study and were ensured about the provision of quitting anytime during the interview. Afterwards, those who gave consent were included. The consent was documented in a form attached with questionnaire by the interviewer.

## Results

Among 3244 participants, majority were male (n = 2300, 70.90%). Out of all, 807 (24.88%) participants were aged more than 46 years, followed by 637 (19.64%) between 26–30 years, 557 (17.17%) between 31–35 years, 496 (15.29%) between 36–40 years, and 320 (9.86%) between 41–45 years. Nearly half (n = 1587, 48.92%) of the respondents were from Dhaka division, and among the respondent 2382 (73.43%) lived in urban areas. Maximum participants completed graduation (n = 1228, 37.85%). However, 381 (11.74%) had either no education or only primary education. More than half of the participants (n = 1714, 52.84%) were service holders, and 322 (9.93%) were healthcare workers (HCW). Most of the study participants’ average monthly family income range was between 20000–40000 BDT (USD $236 - $472) (n = 1260, 41.99%) **(Table 1)**.

**Table 1:** Socio-demographic profile of the study participants.

	Frequency (n)	Percentage (%)
**Age**		
<26	427	13.16
26–30	637	19.64
31–35	557	17.17
36–40	496	15.29
41–45	320	9.86
46+	807	24.88
**Sex**		
Male	2300	70.90
Female	944	29.10
**Division**		
Barisal	125	3.85
Chittagong	477	14.70
Dhaka	1587	48.92
Khulna	221	6.81
Mymensingh	203	6.26
Rajshahi	216	6.66
Rangpur	222	6.84
Sylhet	193	5.95
**Residence**		
Rural	404	12.45
Urban	2382	73.43
Semi-urban	458	14.12
**Religion**		
Muslim	2942	90.69
Non-Muslim	302	9.31
**Educational Status**		
No or primary education	381	11.74
SSC/HSC	1107	34.12
Graduate	1228	37.85
Post-graduate	528	16.28
**Employed**		
Unemployed	122	3.76
Service Holder	1714	52.84
Others	1408	43.40
**Monthly Family Income in BDT($USD)** *		
<20000 (<$236)	746	24.86
20000–40000 ($236 - $472)	1260	41.99
40000–60000 ($472 - $708)	519	17.29
60000+ ($708+)	476	15.86
**Marital status**		
Single	531	16.37
Married	2642	81.44
Divorced/ widowed	71	2.19
**Health care worker**	322	9.93

*Excluding missing values

Approximately one-fourth of our study participants (n = 838, 25.85%) were admitted into the hospital due to COVID-19 infection, while the rest of the patients received treatment in home isolation. Among all, 809 (24.94%) participants were current smokers, and 558 (17.20%) were past smokers. Moreover, 683 (21.05%) participants had hypertension, 621 (19.14%) had diabetes, 492 (15.17%) had asthma or chronic obstructive pulmonary disease (COPD), 354 (10.91%) had heart disease, 212 (6.54%) had chronic kidney disease, and 208 (6.41%) had cancer. The median duration between day of confirmation by RT-PCR and interview was 171 days, ranging from 9 to 266 days **(Table 2).**

**Table 2:** History of hospital admission, smoking status, comorbidity profile and days passed between diagnosis and interview of the respondents.

		Frequency (n)	Percentage (%)
**Admission to the hospital due to COVID-19**		838	25.85
**Smoking status**	**Never smoked**	1877	57.86
	**Current smoker**	809	24.94
	**Past smoker**	558	17.20
**Chronic diseases**	**Hypertension**	683	21.05
	**Diabetes**	621	19.14
	**Asthma/ COPD**	492	15.17
	**Heart disease**	354	10.91
	**Chronic Kidney Disease**	212	6.54
	**Cancer**	208	6.41
**Days passed between diagnosis and interview of the respondents (days), median (IQR)**		171 (131–184)	

The mean score of overall QoL (as assessed by Q1 and scored in a range of 1 to 5) among our participants was 3.50±0.81, which was higher than the middle possible score (i.e., 3). The mean scores of QoL were highest for the physical domain (68.25 ± 14.45) followed by social (65.10 ± 15.78), psychological (63.28 ± 15.48), and environmental domain (62.77 ± 13.07) **(Table 3)**.

**Table 3:** Domain-specific score averages of the study participants.

Domains	Overall mean	Standard deviation
Physical	68.25	14.45
Psychological	63.28	15.48
Social	65.10	15.78
Environmental	62.77	13.07

Physical, psychological, and social domains differed significantly among different age groups (*p* = 0.001) **(Table 4)**. In the physical domain, QoL was found higher in 31–35 years age group, whereas 26–30 year and 41-45year age groups scored higher in the psychological and social domains, respectively (*p*<0.05). Both physical and psychological domain scores among females were statistically significantly lower than males (*p*<0.05 and *p* = 0.001, respectively). In the case of division (level-2 administrative areas of Bangladesh), significant differences in people’s quality of life were observed in all four domains (*p* = 0.001). Physical and psychological domain scores were the highest in Khulna, whereas social and environmental scores were maximum in Sylhet and Barisal divisions, respectively. Participants living in semi-urban areas had a significantly higher physical and psychological domain score, while those living in urban areas had a significantly higher social and environmental domain score. A nearly graded improvement in participants’ quality of life in all four domains was observed with an increase in education (*p* = 0.001). Physical, psychological, and social domain scores were significantly lower in participants who had monthly income BDT <20000 compared to higher-income categories (*p* = 0.001). Employed participant’s quality of life was statistically significantly higher than that of unemployed ones in all four domains except environment (*p* = 0.001). Although single people enjoyed a better quality of life psychologically than those married and divorced, and married participants led a better social life than those who were single, and divorced/widowed. Divorced/widowed persons had a worse physical QoL. Overall, the HCWs enjoyed a better quality of life than the civilians did (*p*<0.05), except in the psychological domain (*p*>0.05). Significantly low QoL was found in physical, psychological, and social domains among the participants who were hospitalized due to COVID-19 infection than those who received home treatments (*p* = 0.001). Current smokers had a significantly low score in all domains of QoL (p<0.05).

**Table 4:** Comparison of individual domain score by socio-demographic variables.

	Physical	Psychological	Social	Environmental
**Age**				
<26	69.65±14.24^ab^	64.99±15.40^ab^	61.42±14.52^c^	62.11±13.68
26–30	70.08±14.24^ab^	65.62±15.77^a^	65.42±16.26^a^	63.41±13.03
31–35	70.49±14.29^a^	64.97±14.67^ab^	66.90±15.89^a^	62.19±13.05
36–40	69.54±13.85^ab^	63.47±15.12^bc^	67.21±16.05^a^	63.48±12.53
41–45	68.47±14.32^b^	61.79±16.04^c^	67.23±15.20^a^	63.07±12.37
46+	63.63±14.26^c^	59.83±15.22^d^	63.42±15.53^b^	62.44±13.36
p	0.001	0.001	0.001	0.322
**Sex**				
Male	68.57±14.55	64.03±15.48	64.89±15.68	62.80±13.06
Female	67.46±14.18	61.44±15.34	65.63±16.02	62.67±13.08
p	0.048	0.001	0.228	0.798
**Division**				
Barisal	69.22±12.92^cd^	55.90±12.63^d^	56.59±11.40^d^	65.92±17.53^a^
Chittagong	64.81±14.95^e^	62.41±14.67^c^	62.26±15.05^c^	62.87±11.93^bc^
Dhaka	67.23±14.36^de^	61.28±15.65^c^	66.43±16.65^b^	63.01±13.05^bc^
Khulna	74.91±14.66^a^	70.49±15.69^a^	66.24±13.26^b^	64.96±11.35^ab^
Mymensingh	66.06±11.59^e^	60.59±13.82^c^	56.14±13.80^d^	59.15±14.39^d^
Rajshahi	72.12±13.14^b^	70.45±16.02^a^	65.08±14.08^b^	62.24±13.58^c^
Rangpur	70.36±14.48^bc^	69.88±12.00^a^	67.35±15.48^b^	61.24±12.52^cd^
Sylhet	72.39±14.25^ab^	65.56±14.21^b^	72.27±12.00^a^	62.07±11.97^c^
p	0.001	0.001	0.001	0.001
**Residence**				
Rural	67.76±14.40^b^	63.82±13.74^b^	61.96±14.56^b^	60.97±12.83^b^
Urban	67.99±14.52^b^	62.69±15.81^b^	65.68±16.03^a^	63.09±13.21^a^
Semi-urban	70.02±14.00^a^	65.85±14.98^a^	64.89±15.15^a^	62.64±12.42^a^
p	0.017	0.001	0.001	0.011
**Religion**				
Muslim	68.30±14.48	63.38±15.55	65.02±15.87	62.64±13.10
Non-Muslim	67.76±14.16	62.25±14.78	65.91±14.87	64.01±12.67
p	0.538	0.228	0.35	0.081
**Educational Status**				
No or primary education	66.11±14.90^b^	60.49±14.07^b^	60.54±16.23^d^	60.65±13.50^c^
SSC/HSC	66.85±14.57^b^	62.99±15.26^a^	62.80±14.78^c^	61.90±13.04^bc^
Graduate	69.19±13.85^a^	64.08±15.21^a^	66.05±15.13^b^	62.91±12.81^b^
Post-graduate	70.52±14.76^a^	64.02±17.24^a^	71.02±16.95^a^	65.76±12.92^a^
p	0.001	0.001	0.001	0.001
**Employment Status**				
Unemployed	63.13±14.20^c^	57.13±13.86^c^	60.72±12.05^c^	64.14±13.47^a^
Employed	70.14±14.02^a^	64.68±15.32^a^	66.53±15.81^a^	63.28±12.81^a^
Others	66.38±14.64^b^	62.11±15.60^b^	63.75±15.85^b^	62.02±13.31^ab^
p	0.001	0.001	0.001	0.014
**Monthly Family Income in BDT($USD)**				
<20000 (<$236)	65.88±14.53^b^	59.96±14.75^c^	59.23±16.54^d^	61.00±15.42^c^
20000–40000 ($236 - $472)	68.27±14.10^a^	64.30±14.66^ab^	64.72±14.45^c^	60.96±11.99^c^
40000–60000 ($472 - $708)	68.90±14.95^a^	65.29±16.96^a^	66.53±16.35^b^	64.70±12.85^b^
60000+ ($708+)	69.45±14.17^a^	63.44±16.50^b^	69.99±15.43^a^	66.72±11.40^a^
p	0.001	0.001	0.001	0.001
**Marital status**				
Single	70.24±14.86^a^	66.32±15.07^a^	61.97±13.83^b^	62.54±12.68
Married	68.07±14.29^a^	62.94±15.43^b^	65.91±16.09^a^	62.86±13.17
Divorced/ widowed	59.99±13.89^b^	53.21±14.97^c^	58.31±13.24^c^	60.77±12.01
p	0.001	0.001	0.001	0.377
**Health care worker**				
No	67.95±14.56	63.34±15.54	64.90±15.84	62.68±13.17
Yes	70.91±13.07	62.68±14.99	66.93±15.14	63.56±12.11
p	0.001	0.466	0.029	0.249
**Admission to the hospital due to COVID-19**				
No	70.03±14.35	65.05±14.92	66.65±15.74	62.65±12.57
Yes	63.16±13.51	58.22±15.99	60.71±15.07	63.13±14.41
p	0.001	0.001	0.001	0.355
**Smoking status**				
Never smoker	69.03±13.99^a^	63.63±14.65^b^	66.95±15.74^a^	62.56±12.82^b^
Current smoker	65.66±14.34^b^	59.49±15.88^c^	61.06±15.50^c^	62.38±14.25^b^
Past smoker	69.34±15.64^a^	67.57±16.34^a^	64.75±15.21^b^	64.03±11.98^a^
p	0.001	0.001	0.001	0.041

Scores were expressed as mean ±SD

^a-d^Scores with different superscript letters have a statistically significant difference across categories of the variable within a domain, e.g., values with a superscript ‘a’ is significantly different from values with other superscript(s).

P-value was determined using one-way ANOVA with post-hoc analysis by Duncan multiple range test.

**Fig 1:**
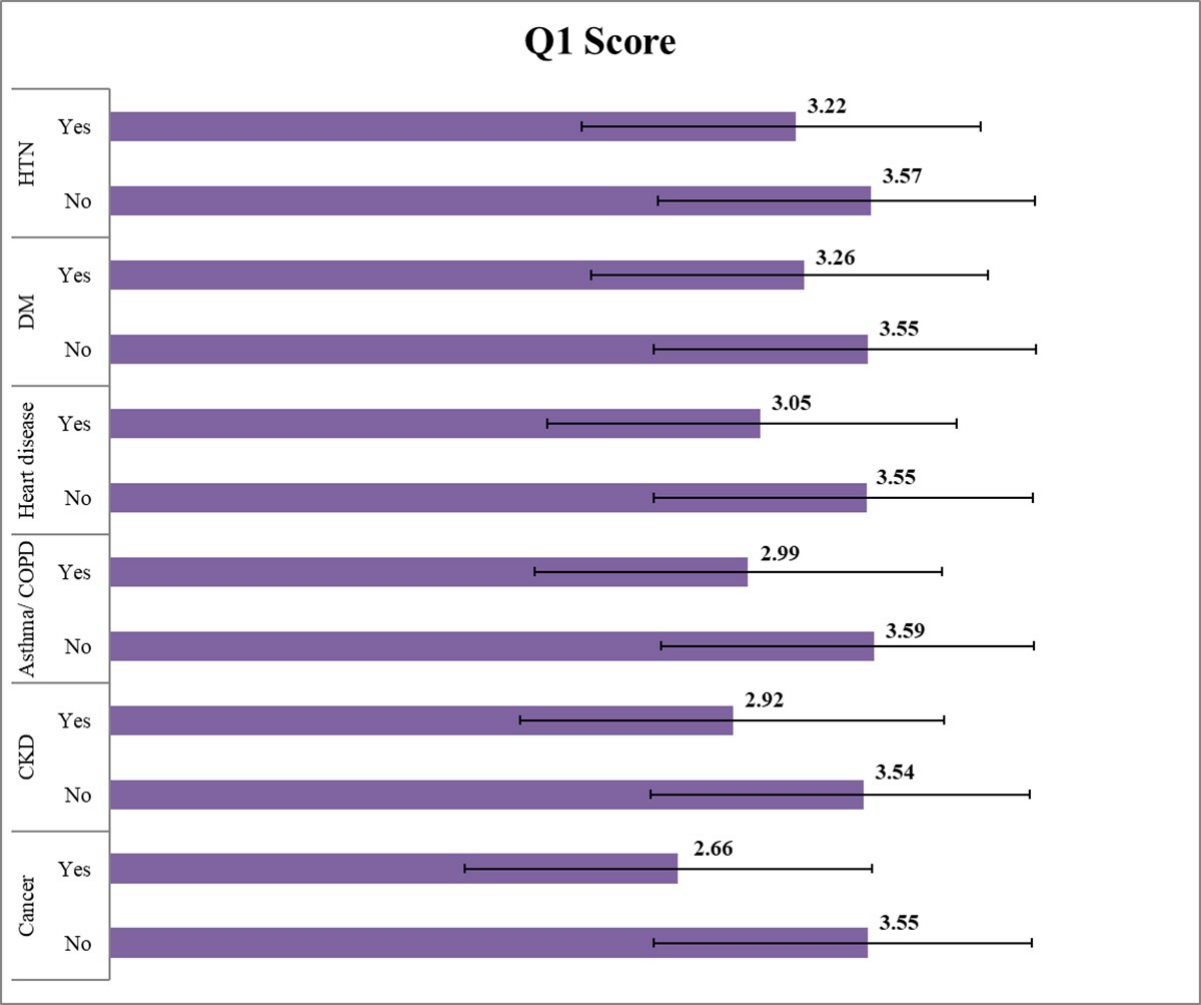
Overall quality of life (Q1 of WHOQOL-BREF) and chronic disease.

The overall quality of life, as assessed by Q1 of the WHOQOL-BREF scale, was lower in persons having a chronic disease than those who didn’t. Among them, COVID-recovered people having cancer had the lowest QoL score **(Fig 1)**.

Mean physical, psychological, social, and environmental domain scores were significantly lower in COVID-19 recovered persons having a chronic disease (hypertension, diabetes, heart disease, asthma or COPD, CKD, and cancer) compared to those who hadn’t(*p*<0.05). The only exception was observed in the environmental domain, which was statistically similar in relation to the presence of heart disease (*p* = 0.464) **(Table 5)**.

**Table 5:** Comparison of individual domain score by chronic disease status.

	Physical	Psychological	Social	Environmental
**Hypertension**				
No	70.00±14.24	65.01±14.94	66.19±15.66	63.07±12.83
Yes	61.66±13.29	56.78±15.78	61.03±15.56	61.63±13.88
p	0.001	0.001	0.001	0.011
**Diabetes**				
No	69.93±14.23	64.89±15.09	66.08±15.62	62.53±12.62
Yes	61.13±13.14	56.46±15.28	60.98±15.78	63.77±14.8
p	0.001	0.001	0.001	0.034
**Heart disease**				
No	69.45±14.36	64.3±15.25	66.10±15.65	62.82±12.74
Yes	58.43±11.02	54.94±14.9	56.97±14.43	62.29±15.50
p	0.001	0.001	0.001	0.464
**Asthma/ COPD**				
No	69.95±14.32	64.99±14.92	66.65±15.30	63.14±12.60
Yes	58.73±11.04	53.67±15.10	56.44±15.63	60.68±15.25
p	0.001	0.001	0.001	0.001
**CKD**				
No	68.84±14.58	63.95±15.19	65.89±15.66	63.14±13.03
Yes	59.72±8.77	53.62±16.37	53.86±12.98	57.47±12.44
p	0.001	0.001	0.001	0.001
**Cancer**				
No	68.9±14.58	64.10±15.19	66.04±15.52	62.85±12.67
Yes	58.63±7.21	51.30±14.85	51.50±12.98	61.57±17.86
p	0.001	0.001	0.001	0.0173

P-values were determined using independent sample t test

We performed a multivariable logistic regression analysis to identify the factors associated with QoL. The results of the analysis are summarized in **Table 6**. We analyzed each variable against each domain to outline the leading associations among them. We observed that a proportionate deterioration of the QoL index in the physical domain occurred with an increasing age. Most significantly, the participants over 46 years of age were 52%less likely (Adjusted Odds Ratio [AOR]: 0.48, 95% Confidence Interval [CI]: 0.28–0.82) to enjoy good physical health than the participants whose age was below 26 years. Psychologically, females were 31% less likely (AOR: 0.69, 95%CI: 0.55–0.88) to have a good quality of life than males. Urban peoples’ chance of having a good physical and psychological quality of life was 37% and 56% lower than the rural people, respectively (AOR:0.63, 95%CI 0.41–0.98, and AOR:0.44, 95%CI 0.29–0.65, respectively). While semi-urban people were 42% less likely to have a good psychological domain score than the rural people (AOR: 0.58, 95%CI: 0.36–0.93). The participants who completed graduation had a 1.65 (95%CI: 1.00–2.57) times higher chance of having a good physical QoL than those without education or with primary education. Also, those who completed graduation and postgraduation had, respectively, 1.45 (95%CI: 1.05–2.01) and1.82 (95%CI: 1.21–2.73) times better social life than those with no education/primary education. Psychologically, service holders’ quality of life was found 1.80 (95%CI: 1.11–2.91) times higher than the unemployed respondents. We observed a nearly proportional increase of QoL in each domain with the rise of the answerer’s monthly income but a consistent and significant decrease with the increasing number of chronic diseases among participants (p<0.05). Likewise, those who were admitted to the hospital during infection were significantly less likely to have a good QoL score in physical, psychological, and social domains than those who weren’t (p<0.05). Past smokers had significantly higher chance of having good QoL in all domains (p<0.05) and current smokers had 28% lower chance (AOR:0.72, 95%CI 0.56–0.93) of maintaining a good psychological QoL. Lastly, for each day passed since recovery, persons with COVID-19 were significantly more likely to have good physical, social and environmental QoL but significantly less likely to have good psychological QoL (p<0.05).

**Table 6:** Factors associated with each domain of WHOQOL-BREF among the study participants in multivariable logistic regression analysis.

	Physical		Psychological		Social		Environmental	
	Adj OR	95% CI	Adj OR	95% CI	Adj OR	95% CI	Adj OR	95% CI
**Age**								
<26	1.00		1.00		1.00		1.00	
26–30	0.72	0.44–1.19	0.95	0.62–1.45	0.93	0.64–1.33	1.18	0.82–1.68
31–35	0.60	0.35–1.05	1.03	0.65–1.66	1.05	0.69–1.59	0.86	0.58–1.28
36–40	0.89	0.49–1.65	1.02	0.63–1.66	1.39	0.69–1.59	1.28	0.68–1.69
41–45	0.63	0.34–1.18	0.95	0.57–1.59	1.20	0.89–2.19	1.07	0.68–1.69
46+	0.48*	0.28–0.82	0.90	0.57–1.41	1.08	0.72–1.62	0.95	0.64–1.41
**Sex**								
Male	1.00		1.00		1.00		1.00	
Female	1.01	0.76–1.32	0.69*	0.55–0.88	0.87	0.71–1.12	1.20	0.96–1.51
**Residence**								
Rural	1.00		1.00		1.00		1.00	
Urban	0.63*	0.41–0.98	0.44*	0.29–0.65	1.19	0.88–1.64	0.98	0.72–1.32
Semi-urban	0.61	0.36–1.03	0.58*	0.36–0.93	1.01	0.69–1.47	1.14	0.78–1.65
**Religion**								
Muslim	1.00		1.00		1.00		1.00	
Non-Muslim	1.13	0.72–1.76	1.02	0.70–1.49	1.14	0.79–1.64	1.29	0.90–1.83
**Educational Status**								
No or primary education	1.00		1.00		1.00		1.00	
SSC/HSC	1.06	0.74–1.52	1.26	0.91–1.76	1.05	0.77–1.42	0.78	0.57–1.06
Graduate	1.65*	1.00–2.57	1.39	0.98–1.98	1.45*	1.05–2.01	0.81	0.59–1.12
Post graduate	1.61	1.00–2.58	1.31	0.87–1.97	1.82*	1.21–2.73	1.04	0.70–1.55
**Employment Status**								
Unemployed	1.00		1.00		1.00		1.00	
Employed	1.17	0.67–2.02	1.80*	1.11–2.91	0.94	0.57–1.54	0.68	0.40–1.14
Others	0.81	0.47–1.39	1.46	0.91–2.34	0.86	0.53–1.40	0.49*	0.29–0.83
**Monthly Family Income in BDT ($USD)**								
<20000 (<$236)	1.00		1.00		1.00		1.00	
20000–40000 ($236 - $472)	1.55*	1.15–2.09	2.03*	1.54–2.67	3.01*	2.36–3.85	1.85*	1.47–2.33
40000–60000 ($472 - $708)	1.31	0.92–1.89	1.65*	1.18–2.29	2.34*	1.73–3.17	3.20*	2.35–4.35
60000+ ($708+)	2.02*	1.34–3.06	1.34	0.96–1.88	3.37*	2.40–4.74	5.65*	3.94–8.11
**Marital status**								
Single	1.00		1.00		1.00		1.00	
Married	1.39	0.90–2.17	0.83	0.56–1.23	0.95	0.67–1.34	1.04	0.76–1.45
Divorced/ widowed	1.32	0.62–2.81	0.46	0.24–0.90	1.07	0.55–2.08	1.21	0.61–2.41
**Health care worker**								
No	1.00		1.00		1.00		1.00	
Yes	1.32	0.80–2.18	1.02	0.71–1.46	0.95	0.67–1.34	0.94	0.67–1.32
**Admission to hospital due to COVID-19**								
No	1.00		1.00		1.00		1.00	
Yes	0.53*	0.41–0.69	0.67*	0.53–0.85	0.68*	0.55–0.86	0.94	0.67–1.32
**Smoking status**								
Never smoker	1.00		1.00		1.00		1.00	
Current smoker	1.08	0.80–1.45	0.72*	0.56–0.93	0.91	0.71–1.15	1.08	0.85–1.35
Past smoker	1.77*	1.22–2.58	1.70*	1.88–2.44	1.56*	1.14–2.13	3.67*	2.60–5.18
**Chronic disease**								
0	1.00		1.00		1.00		1.00	
1	0.51*	0.37–0.70	0.63*	0.48–0.83	0.61*	0.47–0.79	0.93	0.72–1.16
2	0.39*	0.26–0.59	0.36*	0.25–0.53	0.50*	0.34–0.74	0.90	0.60–1.34
3+	0.31*	0.21–0.44	0.18*	0.13–0.25	0.19*	0.14–0.26	0.31*	0.23–0.43
**Duration**^**†**^ **(days)**	1.009*	1.007–1.011	0.997*	0.995–0.999	1.002*	1.000–1.003	1.002*	1.001–1.004

*p <0.05

^†^The time (in days) passed between confirmation of COVID-19 and the date of interview.

**Fig 2:**
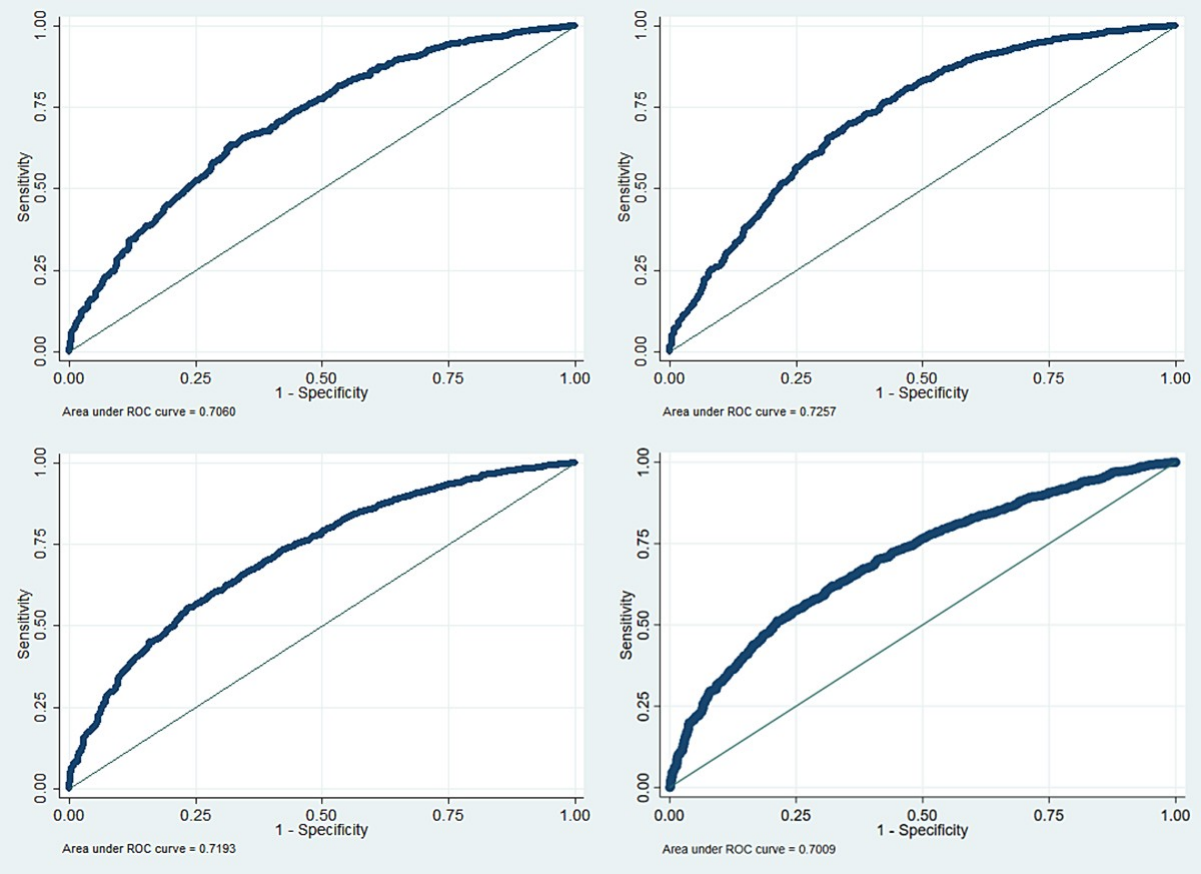
Receiver Operating Characteristic (ROC) analysis of the multivariate logistic regression models.

The overall logit model had a good ability to distinguish between good and poor domain scores **(Fig 2**).

The area under the curve (AUC) of the ROC curve was calculated as a measure of model performance of physical (upper left), psychological (upper right), social (lower left), and environmental (lower right) domains, which explains the model’s performance by evaluating sensitivity versus specificity. There is a 73.13%, 74.29%, 75.34%, and 71.43% chance that the final fitted model is able to distinguish between the positive and negative categories of physical, psychological, social, and environmental scores respectively.

## Discussion

The COVID-19 pandemic has created havoc in the world. It stripped away millions of lives and devastated billions of people economically and psychologically. Those who contracted COVID-19 had to endure the highest suffering. Therefore, we aimed to assess QoL of people who recovered from this disease. Our study reports primary findings on quality of life (QoL) among the largest nationally representative sample of COVID-19 recovered people.

Overall, participants’ perception about their quality of life was just above neutral (Q1 3.5±0.81). This trend was also reflected in the four domains of QoL explored in our study. The mean values of physical, psychological, social, and environmental domain scores were between 60 to 70, indicating an average score at the upper half of the total. The physical domain had the highest average score, followed by social relationship, psychological, and environmental domains, in decreasing order. Nevertheless, these scores indicate an overall good QoL. The initial finding is comparable to the health-related quality of life (HRQoL) of COVID-19 patients after recovery in China, assessed at the beginning of the pandemic. The study conducted by Chen *et al*. [12] found that COVID-19 patients’ average physical component score was just above median (55.96 ±7.24), and psychological domain score was just below median (48.92 ±10.81) one-month after recovery. However, they also noted, both domain scores were significantly lower compared to population norms. The authors used the Short-Form 36-item (SF-36) questionnaire [18], which measures predominantly HRQoL while the WHOQOL-BREF score tends to measure global QoL [19]. Similarly, Jacobs *et al*. [20] reported a low QoL score of COVID-19 patients 35 days after hospitalization, measured by PROMIS® instrument. A comparison of severely affected COVID-19 patients’ QoL in Spain, before and after infection, revealed a statistically significant reduction in QoL at six months [21]. All these studies suggest a considerable change in the quality of life of COVID-19 patients.

Although the overall QoL scores in four domains were good in our study, they showed significant variation in relation to different socio-demographic variables and presence of chronic disease. On multivariable regression male sex, urban residence, higher educational status, being employed (i.e., occupied), being healthcare worker, having a higher income, past smoking habit (smoking cessation), absence of chronic diseases and times passed since recovery were found to be significantly positively associated with a good score of one or more domains of QoL. Similar variations were observed among COVID-19 patients in Saudi Arabia [10] and China [12] and general people in Iran [9] during the middle of the pandemic. Similar to our study, Algahtani *et al*. [10] noted that COVID-19 patients who had chronic disease were significantly more likely have their QoL scores at lowest quartiles. However, unlike our study they noted an association of female sex with a good QoL scores (highest quartiles). While Che *et al*. [12] found that female sex was negatively associated with physical function, bodily pain and role-emotional scores of HRQoL. On the other hand, Arab-Zozani *et al*. [9] noted that unemployed participants were more likely to have a lower QoL score measured by EQ-5D-5L instrument, which is concordant with our findings.

We found that the possibility of having a good physical score decreased with increasing age. However, the psychological, social, and environmental domains remained unaffected by age when adjusted for other factors. As older adults are more likely to suffer from multiple health disorders, their physical QoL is expected to decrease [22], and more so on the top of COVID-19 [9]. Another possible explanation could be the slight increase in the risk of severity of COVID-19 due to increasing age [23]. But that alone might not be sufficient as ‘presence of chronic disease’ and ‘admission to hospital’ were adjusted in the regression model. This implies aging itself posed some negative impact on physical domain scores among COVID-19 patients.

Regarding sex, we found that only psychological domain scores were affected. Being female was associated with a 31% reduction in the chance of having a good psychological score among the participants. In accordance with our result, previous studies have demonstrated similar associations in normal adults of Bangladesh [15], among COVID-19 patients in Iran [9], and one month after COVID-19 infection in China [12]. This might be due to a general decline in women’s mental health during midlife in low and middle-income countries [24]. Added on that a compound economic impact of COVID-19 on women [25] might also have affected them psychologically.

Participants living in urban areas were significantly less likely to have a good physical and psychological domain score. Additionally, those living in semi-urban areas were less likely to have a good psychological score. Rural-urban differences in QoL are multifactorial and vary from region to region. In the developed world, rural areas have much lower QoL than urban areas [26]. This could be true for developing countries, but a high population density and pollution levels in the urban areas might also negatively affect the subjective QoL in these regions [27]. Besides, a lack of green space can trigger a decline in health in urban areas [27]. This also makes sense when we consider that our subjects recovered from a highly infectious airborne respiratory disease.

A general increase in QoL scores in all domains was noted with an increasing level of education, including a significant increase in physical and social relationship domains. This finding supports the work of Skevington [28], who assessed the association of QoL with education in thirteen different countries among both sick and healthy people. He noted that higher educational attainment is generally linked to better occupational prospects and higher income, hence having a positive effect on a person’s quality of life. This finding is also supported by our observation that service holders were significantly more likely to have good psychological QoL than the unemployed participants. During the aftermath of COVID-19, higher-educated people might have maintained personal and social links better than others within the economic and social constraints of the new reality.

We also noted a significant positive association of monthly family income with physical, social and environmental domains of QoL of the participants. But the chance of having a good psychological domain score showed a rise in the middle-income categories with a subsequent decline in higher-income categories among COVID-19 patients. Physical QoL significantly increases with income category [29] possibly because it increases affordability, imparts a sense of security, and is generally associated with higher education. However, subjective well-being is not linearly associated with income and is often dependent on a complex interaction of socio-cultural and environmental factors [30]. This might explain why we saw a decrease in the psychological domain of QoL among COVID-19 recovered participants who had a high monthly family income.

We observed that participants who were admitted in the hospital due to COVID-19 had a significantly lower score in the physical, psychological, and social relationship domain. This points to a possible persistence of symptoms, delayed recovery, and the patients’ stress response after having a severe COVID-19 infection. This was also reflected in the study by Taboada *et al*. [21], who assessed the QoL in COVID patients six months after hospitalization in ICU. Jacobs *et al*. [20] also showed that COVID-19 symptoms could persist up to 35 days impacting the quality of life and mental function.

A striking observation to emerge from the data is that smoking quitters (past smokers) had a significantly better QoL in all domain than non-smokers, and for that matter current smokers. Similarly, current smokers had significantly poor psychological QoL than non-smokers. Studies conducted across populations around the world has consistently reported significant improvement of QoL after smoking cessation [31]. Compared to current smokers, quitters show improved positive mood and mental health [32]. In the setting of COVID-19, ex-smokers’ increased self-esteem, developed from their prior experience of behavioral change, might have drove them to an improved sense of well-being compared to never-smokers. However, we cannot exclude the possibility of unmeasured confounders influencing our analysis. Hence, a cautious reading is requested.

One of the important findings of our study was the association of chronic diseases with a substantially low QoL in all domains. Moreover, the more the number of comorbidities, the less was the chance of having a good QoL. We explored the effect of HTN, DM, heart disease, Asthma/COPD, CKD, and cancer among participants’ QoL. On univariate analysis, the overall domain scores were found significantly lower among those having a chronic disease. But compared to other comorbidities, the Q1 score was above the median in persons having HTN and diabetes, and poor in persons having Asthma/COPD, CKD, and cancer. All of these findings are supported by previous studies conducted among non-COVID persons [33–36]. Besides, we know that chronic diseases in COVID-19 patients can lead to increased disease severity, poor prognosis, and increased mortality [37]. Hence the combined effect of COVID and chronic disease resulted in the lowest quality of life among our participants.

Finally, the most important finding of our analysis was the improvement of the physical, social and environmental QoL of COVID-19 recovered patients with time except psychological domain which endured the highest impact from this devastating disease. Previous reports have shown that nearly one-third person suffered psychological consequences during the current pandemic [8] and our study suggests a sustained decline in mental health of the infected persons after recovery. This warrants further investigation of the matter and urges necessary steps be taken early to prevent further decline in psychological well-being of patients recovered from COVID-19.

Our study was strong in that we took a large randomized sample from eight divisions of Bangladesh representing people from all sides of the country. All of the participants were interviewed after consent over the telephone by trained public health physicians. In addition, we used the WHOQOL-BREF scale to assess quality of life which addresses nearly all domains of life.

The limitation of our study was the lack of a comparison group unaffected by COVID. We couldn’t compare the QoL of patients before and after COVID due to the study’s cross-sectional nature. Nevertheless, we presented QoL measures in a quantitative scale which should be useful for future references.

## Conclusion

COVID-19 patients’ QoL, after recovery, depended on variable interaction of demographic, socio-economic and comorbidity factors. Particularly old age, female sex, low education, unemployment, low monthly income, high disease severity and presence of comorbidity were significantly associated with lower QoL in one or more domains. However, all domains of QoL except the psychological one improved over the passage of time. Our findings would certainly spark interest among national and international communities of researchers, and guide policy makers in developing specific recovery and rehabilitation plans, programs and policies for COVID-19 affected people.

## Supporting information

S1 File Post-COVID QoL in Bangladesh datafile. A Stata file containing curated data of the study. The data file can be used with permission from the authors.
(DTA)
